# Identification of the Geographic Origin of Parmigiano Reggiano (P.D.O.) Cheeses Deploying Non-Targeted Mass Spectrometry and Chemometrics

**DOI:** 10.3390/foods6020013

**Published:** 2017-02-16

**Authors:** Bert Popping, Emiliano De Dominicis, Mario Dante, Marco Nocetti

**Affiliations:** 1 Food Consulting Services, 2 Chemin des Mouilles, 69290 Grezieu la Varenne, France; 2 R&D Department, Italy Mérieux NutriSciences, via Fratta 25, 31023 Resana, Italy; emiliano.de.dominicis@mxns.com (E.D.D.); mario.dante@mxns.com (M.D.); 3 R&D Department, Parmigiano Reggiano Cheese Consortium, Via J. F. Kennedy 18, 42124 Reggio Emilia, Italy; nocetti@parmigianoreggiano.it

**Keywords:** P.D.O., Parmigiano Reggiano cheese, geographical origin, non-targeted mass spectrometry, chemometrics

## Abstract

Parmigiano Reggiano is an Italian product with a protected designation of origin (P.D.O.). It is an aged hard cheese made from raw milk. P.D.O. products are protected by European regulations. Approximately 3 million wheels are produced each year, and the product attracts a relevant premium price due to its quality and all around the world well known typicity. Due to the high demand that exceeds the production, several fraudulent products can be found on the market. The rate of fraud is estimated between 20% and 40%, the latter predominantly in the grated form. We have developed a non-target method based on Liquid Chomatography-High Resolution Mass Spectrometry (LC-HRMS) that allows the discrimination of Parmigiano Reggiano from non-authentic products with milk from different geographical origins or products, where other aspects of the production process do not comply with the rules laid down in the production specifications for Parmeggiano Reggiano. Based on a database created with authentic samples provided by the Consortium of Parmigiano Reggiano Cheese, a reliable classification model was built. The overall classification capabilities of this non-targeted method was verified on 32 grated cheese samples. The classification was 87.5% accurate.

## 1. Introduction

The United States Pharmacopeial Convention (USP) Food Fraud Database [1] carries no less than 474 entries on milk and milk products for the years 2000 to 2015, and milk product adulteration and/or misrepresentation is the second most frequently reported issue.

European legislation prohibits the misrepresentation of products. Beyond the general food safety regulation of the European Commission (EC) 178/2002 [2], the European Commission has implemented a regulation that is commonly known as Consumer Information Regulation EC 1169/2011 [3]. One of the important aspects of this regulation is to provide information to the final consumer that is not misleading. In the reasoning for this regulation (the “whereas” section) it states: “Food information law should prohibit the use of information that would mislead the consumer in particular as to the characteristics of the food, food effects or properties, or attribute medicinal properties to foods. To be effective, that prohibition should also apply to the advertising and presentation of foods.”

In addition to this regulation, Europe has a specific regulation for certain premium products. In its latest regulation, EC 1151/2012 [4], superseding the earlier regulation, EC 510/2006 [5] on quality schemes for agricultural products and foodstuffs, the European Commission clearly defines in Article 12 the use of the P.D.O. (Protected Designated Origin) label. Currently, 600 products carry the P.D.O. label, of which 186 are cheeses, and 49 are from Italy. Parmigiano Reggiano cheese was first registered in 1996. The geographic area in which it can be produced and labeled as P.D.O. is limited to Parma, Reggio Emilia, Modena, and parts of the provinces of Mantua and Bologna, on the plains, hills, and mountains between the rivers Po and Reno. Cattle whose milk is used for the production of Parmigiano cannot be fed silage or fermented feeds, and no additives or preservatives can be used in the cheese production process. To protect P.D.O. products, like Parmigiano Reggiano cheese, the European Commission has bilateral agreements with some countries. There is no such agreement with the United States, which is why certain products can be found there labeled as Parmesan (a term that can only be used inside the European Union and is an acronym for Parmigiano Reggiano), but is not produced under the strict provisions laid down in regulation EC 1151/2012.

To have an additional appropriate tool to identify whether a cheese labeled as Parmiggiano Reggiano is compliant with the P.D.O. definition of the aforementioned regulation, a metabolomics-oriented, non-targeted method that assesses 18 compounds using Liquid Chomatography-High Resolution Mass Spectrometry (LC-HRMS) was developed [6,7,8]. The model was, however, not envisioned to be able to discriminate between samples of cheese produced with milk from the same region (Northern Italy), e.g., Biraghi or Grana Padano. These products are nevertheless easy to be recognized using other methodologies, e.g., single element analysis.

## 2. Materials and Methods

All chemicals were of analytical reagent grade. Acetonitrile and water used as eluents were of Ultra High Pressure Liquid Chromatography–Mass Spectrometry (UHPLC–MS) grade and were purchased from Sigma-Aldrich (Milan, Italy). Formic acid used as additive in eluents and triflumuron used as internal standard was also purchased from Sigma-Aldrich (Milan, Italy). Pierce LTQ Velos ESI Negative ion calibration solutions from Thermo Fisher Scientific (Rockford, IL, USA) were used to calibrate the mass spectrometer.

Authentic samples for reference database generation (52) and blind samples for verification/validation model (32) were provided by Parmigiano Reggiano Cheese Consortium.

The experimental design took into account natural variabilities of “Parmigiano Reggiano grated cheese” production by using an internal standard and analysis of samples in triplicate.

Sample extraction was performed in triplicate with an acidic water/acetonitrile solution by mechanical shaking for 90 min. After centrifugation and defatting, the supernatant was used, and triflumuron was added as internal standard.

UHPLC–MS was performed using a Thermo Scientific Accela 1250 Pump system coupled to a Thermo Scientific Exactive Mass Spectrometer—Orbitrap Technology (Thermo Scientific, Fremont, CA, USA).

UHPLC separation was carried out on a Kinetex XB-C18 (100 mm × 3 mm, 2.6 um particle size) (Phenomenex, CA, USA) using a gradient solvent elution system composed by: ((A) 0.2% *v*/*v* formic acid in water; (B) 0.2% *v*/*v* formic acid in acetonitrile). Gradient elution was as follows: Solvent B was initially set at 0%, then delivered by a linear gradient from 0% to 100% in 13 min. Solvent B was maintained at 100% for 2 min before column re-equilibration (4 min). The flow rate was 0.5 mL/min, and the injection volume was 5 µL.

The Exactive was equipped with Heated-Electrospray Ionization (H-ESI) source with the following settings: sheath gas (N_2_) and auxiliary gas (N_2_) respectively at flow rates of 10 and 5 arbitrary units; a spray voltage of −2.6 kV; a capillary temperature of 275 °C; a capillary voltage of 35; a tube lens voltage of 120; a skimmer voltage of 16.

LC-HRMS analysis was performed in negative polarization mode (100,000 Full Width at Half Maximum (FWMH) resolution), and the full scan data were acquired from 50 *m*/*z* to 900 *m*/*z*.

The mass check/calibration of Exactive Orbitrap was performed before each single batch of analysis to ensure a working mass accuracy lower than 2 ppm.

**Figure 1:**
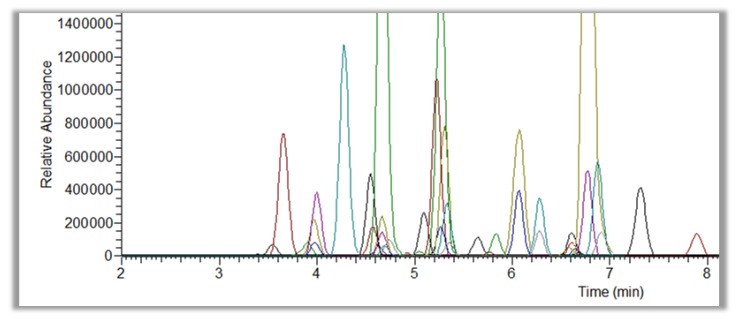
Chromatogram example of Liquid Chomatography-High Resolution Mass Spectrometry (LC-HRMS) Scan 100,000 Full Width at Half Maximum (FWHD) full scan of Parmigiano Reggiano extract (40 peaks are shown in order to demonstrate the raw data quality by means of selectivity, resolution and peak shape).

**Figure 2:**
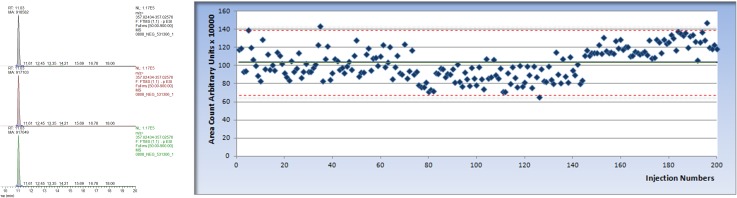
An internal standard with a concentration of 0.5 μg/mL was added to all samples. (**Left**) LC/HRMS chromatogram of specific Extract Ion Chromatogram (EIC) of *m*/*z* values relative to the internal standard; (**Right**) control chart relative to the internal standard used for LC/HRMS in negative mode related to first 200 tests (the black line represents the average of the signal while the red lines show the acceptable levels defined as ±2 Standard Deviation).

A fundamental part of this work was linked to quantifying the normal variability range for relevant parameters that allow for the distinction of authentic Parmigiano Reggiano and fraudulent products. To evaluate this, we took four approaches into consideration:

(1) the inclusion of two quality control samples (pool of 52 reference samples), which were already represented in the database, allowing us to dynamically monitor and verify variations (see Figure 1);
(2) matrix-matched internal standard calibration for the quantitative evaluation of each compound;
(3) the analysis of each sample in triplicate (including three individual extractions) to compensate for extraction variability;
(4) the use of an internal standard to minimize the instrumental variability during the acquisition phase (see Figure 2).

All raw data (peak area compounds normalized through the use of internal standard) were extracted with XCalibur 1.1 software (hermo Scientific, Fremont, CA, USA) (by extracting the accurate mass trace with accuracy <5 ppm) and subjected to statistical analysis. Significant signals (peak area compounds normalized through the use of internal standard scaled with Pareto Scale mode) were then processed with SIEVE 2.0 (hermo Scientific, Fremont, CA, USA) linked to SIMCA 14.0 (mks Data Analytics Solutions, Malmö, Sweden) in order to generate an optimal reference model (Principal Component Analysis (PCA) Class Method), disregarding non-significant signals. Criteria used in this approach to differentiate significant and non-significant signals are extensively described in the literature [6,7,8].

## 3. Results

**Figure 3:**
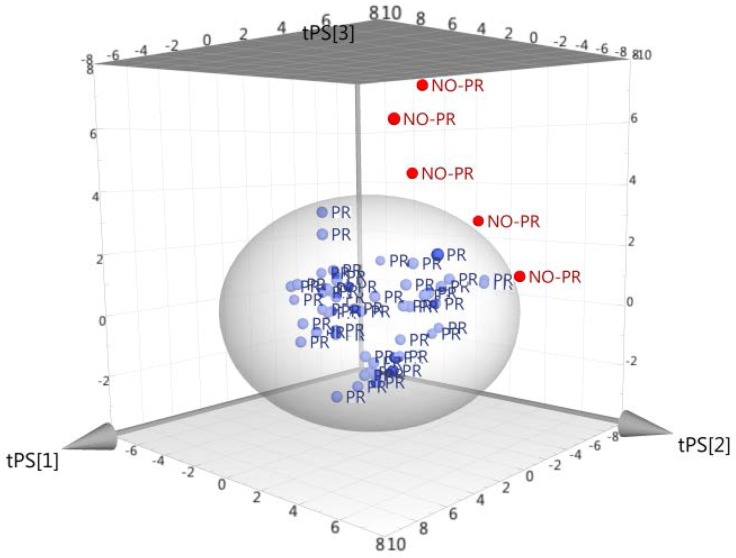
Five grated cheese samples correctly recognized as non-authentic Parmigiano Reggiano (red colored dots, labeled as “NO-PR”; blue colored dots are authentic Parmigiano Reggiano, labeled as “PR”).

A total of 52 samples used to train the model. All were extracted using the above-described method. The extracts including the internal standard were analyzed on the Thermo Scientific Exactive Mass Spectrometer using full scan mode (100,000 FWMH resolution). The full scan (see Figure 3) was applied to obtain the information as to which molecules could be used as differentiator for authentic and fraudulent products.

The chemometric model generated and verified (PCA Class Method) was built successively so that samples known to be authentic Parmigiano Reggiano (as verified by the Parmigiano Reggiano Consortium) from the previous analysis were recognized as authentic such. The identified natural variabilities between the authentic samples were incorporated into the recognition system for correction. This continuously improved the classification capabilities of the SIMCA 14.0 software used.

The final generated method (PCA for class) using a total of 52 reference samples, analyzing 6 principal components for presence and quantity, returned an R^2^ (Coefficient of Determination) of 0.972 and a Q^2^ (Cross Validation of Coefficient of Determination) of 0.796. The developed system demonstrated a recognition ability of 87.5%. This was verified using 32 blind samples (with geographical Origin know by Parmigiano Reggiano Consortium but blind/not known by R&D Laboratory of Mérieux Nutrisciences—for details and samples description, see Table 1).

## 4. Discussion

In order to develop reliable untargeted approaches, the experimental design needs to take into account all possible permitted production variables—in this study, for the product “Parmigiano Reggiano Grated Cheese.” This includes, e.g., the degree of ripening. In our model, we included samples with four different levels of maturation: low (10–12 months); low–medium (15 months), medium–high (22–24 months), and high (36 months). We also took into account the amount of crust present in the grated product: for this, we included samples with two different levels of crust: (a) no crust and (b) 15%–18% (the maximum allowed limit of crust according to the production specifications). For fat, we have included samples with three various fat contents: normal (43%–45% on dry matter), low (<43% on dry matter), and high (>45% on dry matter) fat content.

For the development of such accurate prediction models, the in-depth knowledge of such production variability is essential. Building these factors into our model, we could include these and thereby reduce the number of false positive recognitions.

**Figure 4:**
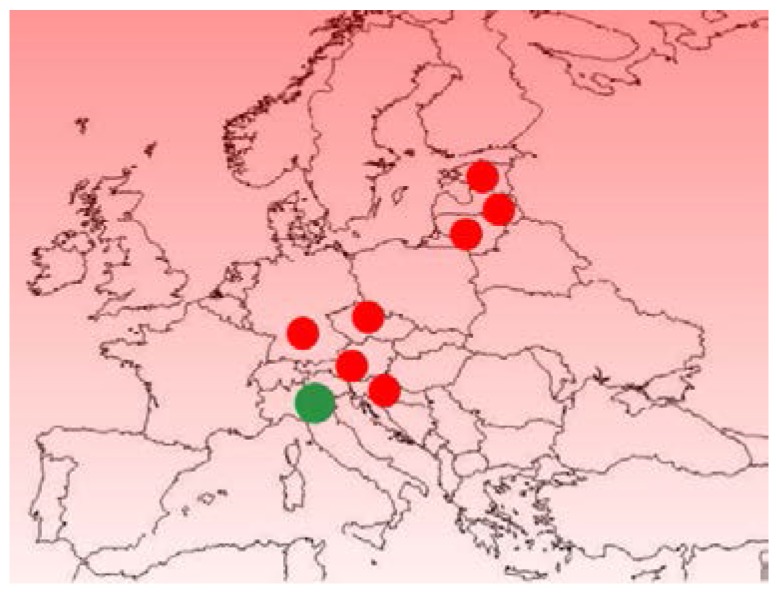
Green circle indicates geographic region of authentic Parmigiano Reggiano production. Red circles indicate cheeses of other geographic origins that the model is able to differentiate from authentic Parmigiano Reggiano.

This model was expected to identify cheeses produced with milk obtained from other geographic origins, in particular those more frequently used to counterfeit Parmigiano Reggiano. The system demonstrated that samples from other geographic locations were correctly predicted to be non-authentic Parmigiano Reggiano (Figure 4).

## 5. Conclusions

To identify fraudulent products presented as Parmigiano Reggiano (P.D.O.) but containing milk from regions other than Northern Italy, this non-targeted method was developed. Gas Chromatography (GC)-based methods for analyzing volatile compounds in Italian parmesan and those cheeses marketed in New Zealand as parmesan have previously been described [9]. However, the goal was to identify the different flavors, not the geographic location based on the metabolomics profile. The LC-based method and subsequent statistical analysis here have been developed to distinguish the geographic origin of the cheese. This method contributes to identify food fraud and counterfeiting. The model has a high prediction accuracy of 87.5%. In order to distinguish also cheeses produced in the same region in a similar way to Parmigiano Reggiano, other parameters can be taken into account to increase the differentiating power of this model. This new method developed based on High Resolution accurate Mass (HRAM) and statistical analysis using SIEVE 2.0 and SIMCA 14.0 has been shown here, with more than 50 samples analyzed, to be robust and reliable across all samples. This method takes permitted variabilities in the production into account, including different quantities of crust, different quantities of fat, and different maturation degrees.

## Figures and Tables

**Table 1:** Classification results. PR = sample compliant with Parmigiano Reggiano specifications according to the statistical model; NO-PR = sample not compliant with Parmigiano Reggiano specifications according to the statistical model; PCA, Principal Component Analysis.

Sample Code	Sample Description	Theoretical Geographical Origin	Expected Classification	(Non Targeted MODEL)—PCA for Class	
				Classification Score	Classification Result
15.539755.0001	Parmigiano Reggiano (12 months)	North Italy	PR	1.15	PR
15.539755.0002	Riebekase	Outside North Italy	NO-PR	2.83 × 10^−15^	NO-PR
15.539755.0003	DzJugas	Outside North Italy	NO-PR	6.66 × 10^−15^	NO-PR
15.539755.0004	Grana Padano	North Italy	NO-PR	2.52 × 10^−4^	NO-PR
15.539755.0005	Parmigiano Reggiano (10 months)	North Italy	PR	1.82	PR
15.539755.0006	Parmigiano Reggiano (12 months)	North Italy	PR	0.91	PR
15.539755.0007	Parmigiano Reggiano (15 months) High Crust	North Italy	PR	0.53	PR
15.539755.0008	Grana Padano	North Italy	NO-PR	0.94	PR
15.539755.0009	Gran Moravia	Outside North Italy	NO-PR	0.07	NO-PR
15.539755.0010	Parmigiano Reggiano (12 months)	North Italy	PR	1.82	PR
15.539755.0011	Parmigiano Reggiano (15 months) High Fat	North Italy	PR	1.21	PR
15.539755.0012	Biraghi	North Italy	NO-PR	1.40	PR
15.539755.0013	Parmigiano Reggiano (12 months) Low Fat	North Italy	PR	1.69	PR
15.539755.0014	Parmigiano Reggiano (22 months)	North Italy	PR	1.11	PR
15.539755.0015	Parmigiano Reggiano (30 months)	North Italy	PR	0.79	PR
15.539755.0016	Parmigiano Reggiano (12 months)	North Italy	PR	1.7	PR
15.544602.0001	Parmigiano Reggiano	North Italy	PR	0.29	PR
15.544602.0002	Hartkase (Ger)	Outside North Italy	NO-PR	3.26 × 10^−21^	NO-PR
15.544602.0003	Grana Padano from Retailer	North Italy	NO-PR	0.20	PR
15.544602.0004	Parmigiano Reggiano	North Italy	PR	0.63	PR
15.544602.0005	Parmigiano Reggiano	North Italy	PR	0.54	PR
15.544602.0006	Parmigiano Reggiano	North Italy	PR	0.60	PR
15.544602.0007	Gran Biraghi	North Italy	NO-PR	0.59	PR
15.544602.0008	Parmigiano Reggiano	North Italy	PR	0.92	PR
15.544602.0009	Goya	Outside North Italy	NO-PR	2.75 × 10^−13^	NO-PR
15.544602.0010	German grated cheese	Outside North Italy	NO-PR	0.16	NO-PR
16.552630.0001	Lituania grated cheese	Outside North Italy	NO-PR	1.35 × 10^−9^	NO-PR
16.552630.0002	Parmigiano Reggiano	North Italy	PR	3.47	PR
16.552630.0003	Lettonia grated cheese	Outside North Italy	NO-PR	1.23 × 10^−3^	NO-PR
16.552630.0004	Lituania + German grated cheese	Outside North Italy	NO-PR	1.77 × 10^−3^	NO-PR
16.552630.0005	Estonia grated cheese	Outside North Italy	NO-PR	1.26 × 10^−14^	NO-PR
16.552630.0006	Parmigiano Reggiano	North Italy	PR	8.16 × 10^3^	PR
